# Catarrh

**Published:** 1881-08

**Authors:** 


### CATARRH

Is a Greek word, which means a "flowing from," and is synonymous with a common cold. A cold in the head causes a running from the nose; a cold in the eyes makes them water; a cold in the chest or lungs causes an increased expectoration; a cold in the bowels occasions diarrhea. This "flowing," whether from nose, eyes, lungs or bowels, is nature's effort to ward off the effects of a previous injury; it is essentially a curative process, and ought never to be interfered with. If this "flowing from" is stopped in any way, whether by external applications or internal medicines, the inevitable effect, always, is to drive it to some other part to seek an outlet, for nature will not rest ever, until the riddance is effected. Within a month, a lady was attacked with a great itching and running in the nose, some ignoramus advised her to use a certain kind of snuff, to "dry it up;" it had the effect in a few hours, and she was charmed with the result; she thought it a wonderful medicine; that night she was attacked with asthma, which confined her to her bed for two weeks, to say nothing of the distressing sufferings which filled the interval, day and night.

A gentleman complained of a cold in the head, with sick headache; some one advised him to have buckets of cold water poured on the top of his head, which was followed by a welcomed relief; the next day he complained of a sore throat, which troubled him as long as he lived.

Many persons have diarrhea as a consequence of a cold; they can not rest until they "take something" to "check it," with the certain result of its falling on the liver, to end in a

"bilious attack," if not on the lungs, to cause pneumonia, or pleurisy, or other more serious form of disease.

A gentleman had a cold in the head which affected his hearing; it was ignorantly tampered with, and apparently cured; but the eyes began to complain shortly after, to remedy which he spent two years and a thousand dollars under the most eminent Allopaths and Water-Cures, with no efficient result; and his eyes are as troublesome to-day as they were some ten years ago. All "flowings," "runnings," etc., are the result of what, in common parlance, is a "humor in the blood," and nature is endeavoring to "run it off," but our reckless and ignorant interferences thwart her in her efforts, and bring on greater calamities.

In all catarrhs, chronic or acute, long or short, a wise physician will do nothing to stop or repress, but will use means to cause a greater activity of the liver, and prescribe an unstimulating and cooling diet, warmth and judicious exercise.

For ourselves we would give physic a wide berth. If we had a "flowing from," a catarrh, a cold, all of which mean precisely the same thing in nature and essence, we would let it flow, and thus have the system relieved of an enemy, whose presence it will not tolerate. But there are three other things which may be done to very great advantage, because they would expedite the cure.

1. Keep the body very comfortably warm by all available means, especially the feet.

2. Take a good deal of exercise in the open air, to the extent of keeping up a very slight perspiration for several hours during the twenty-four.

3. Live on light, loosening, cooling food—moderate amounts—such as water-gruel, crust of bread, stewed fruits, ripe berries, and nothing else, until entirely well.

---

"FISH FOR FOOD.—Fish are said to be a very healthy food With the exception of such as have oil interfused in their muscular tissues, fish are easy of digestion, and it is remarkable that fishermen and their families and those who consume a large quantity of fish, are healthy to a more than ordinary degree, and are almost wholly exempt from scrofula and pulmonary consumption."

